# Development of Neuroleptic Malignant Syndrome in a Patient with Lewy Body Dementia after Intramuscular Administration of Paliperidone

**DOI:** 10.1155/2021/8879333

**Published:** 2021-01-13

**Authors:** Ho-Man Yeung, Sarah Schmitz, Nino Kvantaliani, Christina Martin

**Affiliations:** ^1^Department of Medicine, Lewis Katz School of Medicine at Temple University, Philadelphia, PA, USA; ^2^Department of Neurology, Lewis Katz School of Medicine at Temple University, Philadelphia, PA, USA

## Abstract

Neuroleptic malignant syndrome (NMS) is a potentially fatal diagnosis composed of hyperpyrexia, muscle rigidity, altered mental status, and autonomic instability. This syndrome has significant systemic complications including acute renal failure, rhabdomyolysis, hyperkalemia, and seizure. It is associated with the use of both typical and atypical antipsychotics. Due to the extensive neurodegenerative destruction of dopaminergic and acetylcholinergic pathways, patients with Lewy body dementia (LBD) are particularly sensitive to antidopaminergic and anticholinergic medications, making them more susceptible to extrapyramidal side effects and NMS. We present a case of a 72-year-old female with LBD who developed muscular rigidity, vital sign instability, and altered mental status after receiving one dose of paliperidone palmitate injection two weeks prior to admission. Initial blood work was unrevealing. Extensive workup including EEG, lumbar puncture with cerebrospinal fluid analysis, and brain MRI was unremarkable. She was treated with seven days of bromocriptine and a lorazepam taper with improvement in muscle rigidity. However, her mental status never improved, and she remained comatose. She was later intubated for airway protection after an aspiration event that led to hypoxia. Her respiratory status never recovered, and she was transitioned to comfort care. This case demonstrates the complexity and potential fatality of NMS. Clinicians should be aware of this dangerous complication of antipsychotic use in patients with LBD as these patients may be more susceptible to this complication.

## 1. Introduction

Neuroleptic malignant syndrome (NMS) is a rare complication of antipsychotic use, with an incidence of 1 to 2 cases per 10,000 patients treated with antipsychotics [1]. It is characterized by the tetrad of altered mental status, muscle rigidity, fever, and autonomic instability. The rigidity is typically described as cogwheel and lead-pipe rigidity. It usually occurs shortly after initiation of therapy or after a dose adjustment, often over a period of 1 to 3 days [1]. Common laboratory abnormalities include elevated creatine kinase and leukocytosis; however, neither are specific for the syndrome. NMS is associated with all typical antipsychotics and most atypical antipsychotics including aripiprazole, clozapine, olanzapine, risperidone, and ziprasidone. Notably, fever and autonomic instability have less commonly been reported as features of NMS associated with atypical antipsychotics [1].

Risk factors for the development of NMS are the initiation or increased dosing of an antipsychotic medication, the potency of the drug, and the route of administration of the drug. The use of high-dose, high-potency, or long-acting or intramuscular forms of antipsychotics increases the risk of developing NMS [2]. Other risk factors identified in epidemiological and case studies of NMS include the presence of a structural or functional brain disorder such as tumor, encephalitis, delirium, or dementia [3].

Lewy body dementia (LBD) is characterized by Parkinsonism with visual hallucinations and fluctuating alertness. On microscopic review, Lewy bodies are seen throughout the cerebral cortex, which are often also associated with Alzheimer disease pathology. The prevalence is likely underestimated given the difficulty in making the neuropathologic diagnosis; however, LBD represents about 15% of dementias seen at autopsy [4]. Patients with LBD are highly sensitive to metabolic disturbances, and in some patients, the first manifestation of illness is delirium precipitated by a new medication, infection, or other systemic disturbances. The mainstay of treatment for LBD is cholinesterase inhibitors. If psychosis is a prominent symptom, an atypical antipsychotic can be used. However, they may worsen extrapyramidal symptoms, even at low doses. These medications are associated with an increased risk of death; therefore, they should be used cautiously, especially because patients with LBD are very sensitive to dopaminergic medications [5]. Although there is a significant overlap between the physical findings of Parkinsonism due to LBD and NMS, the two entities can be distinguished by the acuity and severity of rigidity. Furthermore, those with NMS will often experience autonomic instability and hyperthermia.

We present a case of a 72-year-old Puerto Rican female with LBD, hypertension, and type 2 diabetes mellitus who received 1 dose of paliperidone palmitate intramuscular injection and later presented with clinical features of NMS, complicated by aspiration and pneumonia, requiring mechanical ventilation. This case demonstrates the caution that should be taken when treating LBD with atypical antipsychotics, especially in the long-acting injectable form.

## 2. Case Presentation

A 72-year-old Puerto Rican female with a past medical history of advanced dementia with Lewy bodies, type 2 diabetes mellitus, and essential hypertension was brought to the emergency department by her granddaughter with acute change in mental status. At baseline, the patient was nonverbal, but could typically eat, use the restroom independently, and walk without significant assistance. For the past two weeks, she was unable to perform any tasks without full dependence, and she was unable to recognize her family. There was no history of drug or alcohol use. Her home medications included aspirin 81 mg daily, donepezil 10 mg daily, docusate sodium 100 mg daily, losartan 50 mg daily, linaclotide 72 mcg daily, metformin 500 mg twice daily, ranitidine 150 mg twice daily, and simvastatin 40 mg daily. Review of systems per family was negative for fevers, chills, cough, diarrhea, change in urinary frequency, or dysuria.

Lewy body dementia was diagnosed several years prior by a neurologist and had been progressive with urinary incontinence, hallucinations and paranoia, and speech difficulty. Due to her hallucinations and behavioral challenges, her primary care physician had initiated low-dose oral haloperidol. Medication adherence was difficult given her dementia, and she continued oral haloperidol inconsistently for several months. Two weeks prior to admission, her PCP initiated paliperidone palmitate extended-release intramuscular injection of 156 mg, as the patient had been off antipsychotic therapy for three months.

On admission, the patient had a temperature of 97.4°F (36.3°C), a heart rate of 102 bpm, a blood pressure of 130/67 mmHg, and an oxygen saturation of 97% on room air. On examination, the patient responded only to painful stimuli by grimacing. Pupils were equal but sluggishly reactive to light. Her head was deviated to the left and stiff when moved toward the midline. She did not follow any commands or demonstrate any voluntary movements. She had muscular rigidity of her upper extremities and lower extremities bilaterally. Her patellar and brachial reflexes were brisk at 3+, and her toes were mute bilaterally. The following day, she became hyperthermic to 101.8°F (38.8°C) with a blood pressure of 149/88 mmHg. On hospital day 3, her temperature continued to fluctuate between 97.6 and 100.7°F (36.4–38.2°C) with heart rate ranging 89–110 bpm, and her blood pressures ranged 130–172/61–93 mmHg. She was seen by toxicology, psychiatry, and neurology on hospital day 2 and was transferred to the ICU. She was started on dantrolene, bromocriptine, and benzodiazepines for symptomatic management of suspected neuroleptic malignant syndrome.

Basic laboratory workup showed no leukocytosis and no electrolyte abnormalities. See Table 1 for a summary of the workup. Total creatine kinase (CK) was 276 U/L (lab reference range: 26–192 U/L) on admission, which subsequently decreased to 222 U/L and later 205 U/L. Head CT was unremarkable, and EEG showed diffuse slowing. MRI brain without contrast revealed no acute findings. Urinalysis had 0–2 WBCs/HPF and was negative for leukocyte esterase; however, urine culture grew >100k cfu *Enterococcus faecalis*. Given her critical condition, she was initiated on vancomycin and piperacillin/tazobactam without clinical improvement.

Lumbar puncture was performed with normal opening pressure. Cerebrospinal fluid (CSF) was clear in color with 2 WBCs/mm^3^, glucose 92 mg/dL, and protein 27 mg/dL, which was not suggestive of meningitis or encephalitis. CSF smear showed no organisms, and cultures grew no organisms. CSF VDRL was nonreactive. Acid-fast stain and smear showed no acid-fast bacilli. CSF cryptococcus antigen was negative. CSF anti-NMDA receptor antibody was negative. Cytology analysis of the CSF showed no malignant cells. Antibiotics were discontinued on hospital day 5 as blood cultures had no growth.

The patient remained unresponsive, but her rigidity and vital signs improved on hospital day 4. She was afebrile and normotensive. Two days later, the patient became tachypneic and hypoxic, saturating 87% on room air, with suspicion for aspiration. She was intubated for airway protection. A postintubation chest X-ray showed right lower lobe infiltrative changes, for which ampicillin/sulbactam was started. She was also started on amantadine and was titrated off all sedating medications. Unfortunately, she did not have any significant improvement in her mental status and remained mechanically ventilated. She completed a 7-day course of antibiotics for aspiration pneumonia; however, she continued to be apneic on spontaneous breathing trials.

After an ongoing discussion about the patient's grim prognosis, the family decided on palliative extubation and transition to comfort care. The patient was ultimately discharged to inpatient hospice on hospital day 15.

## 3. Discussion

The case described here is the first known case in the English literature of a patient with Lewy body dementia that suffered from neuroleptic malignant syndrome after receiving an intramuscular injection of paliperidone palmitate. Prior to making a diagnosis of NMS, other diagnoses should be excluded. The differential diagnosis includes metabolic, toxicologic, infectious, and endocrine disorders. Neuroleptic malignant syndrome may be difficult to distinguish clinically from serotonin syndrome. Serotonin syndrome generally has a more rapid onset of 2–24 hours, hyperreflexia, myoclonus, nausea, vomiting, diarrhea, and less intense muscle rigidity and fever [1].

Our patient was diagnosed with NMS as she experienced the tetrad of a change in mental status progressing to coma, muscular rigidity, hyperthermia >38°C, and autonomic instability in the setting of recent administration of a long-acting injection of an antipsychotic [6]. There are several confounders in this case. She may have had worsening Parkinsonism from LBD, which may explain the rigidity and elevated CK; however, it would not completely explain the patient's coma, hyperthermia, or autonomic instability. Her urine culture was concerning for a urinary tract infection; however, her urine had very few WBCs on microscopy, contrary to what one would expect from an active infection leading to critical illness. This would suggest colonization rather than infection, especially when the patient did not improve despite broad-spectrum antibiotics. Lastly, the patient's serum CK was mildly elevated. Although the serum CK is typically >1000 U/L in NMS, it is nonspecific for NMS, and normal to mildly elevated CK levels have been reported if the rigidity is nonsevere, in early onset of the disease, or in patients with muscle wasting [7–9].

The mainstay of treatment for NMS is discontinuation of the offending agent and supportive care. External cooling can be utilized to reduce the patient's temperature; however, antipyretics such as acetaminophen have not been shown to be beneficial. Sedation with benzodiazepines is recommended to decrease agitation and sympathetic activity. Those with fever and rigidity may require intubation because neuromuscular paralysis reduces muscle contraction and therefore can reduce fever. Nonpolarizing agents are preferred over depolarizing agents for intubation [1].

Most deaths due to NMS are related to profound muscle rigidity resulting in complications including respiratory failure, rhabdomyolysis, renal failure, disseminated intravascular coagulation, and cardiovascular collapse. No treatment has been shown to be superior to supportive care; however, reported cases have described the use of dantrolene, bromocriptine, benzodiazepines, and amantadine [1]. The mortality rate is around 10%; however, most patients completely recover in 2 to 14 days if NMS is recognized early and treated aggressively. If diagnosis and treatment are delayed, then those who survive may have residual catatonia or Parkinsonism [10].

Paliperidone palmitate is a long-acting formulation of paliperidone, an active metabolite of risperidone. It is an antagonist of brain dopamine D_2_ and serotonin (5-hydroxytryptamine) receptors. Effects are usually seen about 8 days after injection, and peak plasma level is reached about 13 days after injection. The half-life ranges from 25 to 49 days [11]. Intramuscular administration of a neuroleptic may result in higher peak levels and abrupt dopamine blockade. Therefore, paliperidone palmitate should be used with caution in those with LBD as these patients have been reported to have marked sensitivity to this class of medications [2]. Patients with LBD have poor adherence to medications; therefore, accurate supervision by caretakers is crucial for medication optimization [12], before deciding to switch to a different medication or escalate doses. Prior to administering long-acting intramuscular antipsychotic in any patients, the risk-to-benefit ratio with oral agents should be considered first, as their response may be difficult to predict and may be severe, particularly those with LBD. As potential adverse effects from long-acting intramuscular antipsychotics may not be easily reversed, oral medications such as clozapine or quetiapine at a low dose may be appropriate choices to minimize potential extrapyramidal symptoms [13, 14]. Relative to other NMS cases seen with oral agents [15–17], one may speculate that long-acting injections may have longer onset time and have long-lasting side effects due to their delayed peak plasma concentration and long half-lives. Kane et al. reported the incidence of NMS associated with paliperidone palmitate as 4/10,000 patient-years using the Janssen database. Of the 5008 patients enrolled, only 1 case of NMS was identified. Although that patient did not carry a diagnosis of dementia, he received a 50 mg injection of paliperidone palmitate and subsequently suffered NMS on day 14 with full recovery after 32 days [18]. Our case is the first reported case of NMS in a patient with LBD following the administration of paliperidone palmitate. As the use of novel long-acting antipsychotics is still in its nascency, clinicians should be aware of this serious and potentially fatal complication of paliperidone palmitate use, especially in those with underlying dopamine sensitivity.

## Figures and Tables

**Table 1:** Laboratory and radiographic workup.

**Laboratory data**	**Culture data**
Hemoglobin 14.3 g/dL	Blood cultures NGTD
WBC 9.0 × 1000/mm^3^	CSF cultures NGTD
Sodium 139 mEq/L	Urine culture > 100k *E. faecalis*
Potassium 5.2 mEq/L	Sputum culture < 10k *S. aureus*
BUN 19 mg/dL	**Imaging findings**
Creatinine 0.89 mg/dL	Normal CT head
Glucose 193 mg/dL	Normal MRI brain without contrast
LFTs wnl	No seizure activity on EEG
Lactate 1.2 mmol/L	**CSF fluid analysis**
Creatine kinase 276 *μ*/L	Clear color
Ammonia wnl	2 WBCs/*μ*L
TSH 0.970 mU/L	Glucose 92 mg/dL
UDS negative	Protein 27 mg/dL
RPR nonreactive	VDRL nonreactive
Hepatitis C Ab nonreactive	Cryptococcal Ag negative
HIV nonreactive	AFB stain negative
B12 wnl	NMDA receptor Ab negative

WBC: white blood cell; BUN: blood urea nitrogen; LFT: liver function test; TSH: thyroid-stimulating hormone; UDS: urine drug screen; RPR: rapid plasma reagin; NGTD: no growth to date; VDRL: venereal disease research laboratory; AFB: acid-fast bacillus; NMDA: N-methyl-D-aspartate; wnl: within normal limits.
